# Genetic profile in genes associated with muscle injuries and injury etiology in professional soccer players

**DOI:** 10.3389/fgene.2022.1035899

**Published:** 2022-11-16

**Authors:** Antonio Maestro, Juan Del Coso, Millán Aguilar-Navarro, Jorge Gutiérrez-Hellín, Esther Morencos, Gonzalo Revuelta, Eva Ruiz Casares, Teresa Perucho, David Varillas-Delgado

**Affiliations:** ^1^ Faculty of Medicine, Oviedo University, Begoña Hospital, Gijón, Spain; ^2^ Centre for Sport Studies, Faculty of Health Sciences, Rey Juan Carlos University, Madrid, Spain; ^3^ Faculty of Health Sciences, Universidad Francisco de Vitoria, Madrid, Spain; ^4^ Clinica El Molinon, Gijón, Spain; ^5^ VIVOLabs, Madrid, Spain; ^6^ Department of Genetics, Faculty of Medicine, San Pablo-CEU University, Madrid, Spain; ^7^ Faculty of Biological Sciences, Universidad Complutense de Madrid, Madrid, Spain

**Keywords:** muscle performance, single nucleotide polymorphism, genes, injuries, muscle injuries, epidemiology, football (soccer)

## Abstract

Many causes define injuries in professional soccer players. In recent years, the study of genetics in association with injuries has been of great interest. The purpose of this study was to examine the relationship between muscle injury-related genes, injury risk and injury etiology in professional soccer players. In a cross-sectional cohort study, one hundred and twenty-two male professional football players were recruited. *AMPD1* (rs17602729), *ACE* (rs4646994), *ACTN3* (rs1815739), *CKM* (rs8111989) and *MLCK* (rs2849757 and rs2700352) polymorphisms were genotyped by using Single Nucleotide Primer Extension (SNPE). The combined influence of the six polymorphisms studied was calculated using a total genotype score (TGS). A genotype score (GS) of 2 was assigned to the “protective” genotype for injuries, a GS of 1 was assigned to the heterozygous genotype while a GS of 0 was assigned to the “worst” genotype. Injury characteristics and etiology during the 2021/2022 season were classified following a Consensus Statement for injuries recording. The distribution of allelic frequencies in the *AMPD1* and *MLCK* c.37885C>A polymorphisms were different between non-injured and injured soccer players (*p* < 0.001 and *p* = 0.003, respectively). The mean total genotype score (TGS) in non-injured soccer players (57.18 ± 14.43 arbitrary units [a.u.]) was different from that of injured soccer players (51.71 ± 12.82 a.u., *p* = 0.034). There was a TGS cut-off point (45.83 a.u.) to discriminate non-injured from injured soccer players. Players with a TGS beyond this cut-off had an odds ratio of 1.91 (95%CI: 1.14–2.91; *p* = 0.022) to suffer an injury when compared with players with lower TGS. In conclusion, TGS analysis in muscle injury-related genes presented a relationship with professional soccer players at increased risk of injury. Future studies will help to develop this TGS as a potential tool to predict injury risk and perform prevention methodology in this cohort of football players.

## Introduction

Variations in the desoxyribonucleic acid (DNA) sequence have been associated with athletic performance (Pitsiladis et al., 2013), cardiovascular response to training (Zarebska et al., 2014; Varillas-Delgado et al., 2021) and soccer-related phenotypes, such as sprinting and power (Eynon et al., 2013; Murtagh et al., 2020), and jumping capacity (Massidda et al., 2012; Massidda et al., 2014). This genetic information can be used to create individualized training programs based on a player’s genetic predispositions, which may represent a revolution in current methods of training. Recently, an association has been shown between a few single nucleotide polymorphisms and the susceptibility to develop musculoskeletal injuries and anterior cruciate ligament rupture in professional soccer players (Pruna et al., 2013; Massidda, Bachis, et al., 2015a; Massidda, Eynon, et al., 2015b; Massidda et al., 2019; Massidda et al., 2020). The identification of genetic variants that predispose athletes to a higher/lower risk of injury is of interest to coaches, physiologists and the medical community, and will help them to optimize the detection of sports talents (Varillas-Delgado et al., 2022a).

Injury etiology is related to the kind of sport model. In this regard, the presence of allelic differences in candidate genes related to sports performance (Sessa et al., 2011) has been reported in power sports (sprinters, short-distance swimmers, and volleyball) and intermittent sports (soccer, basketball, and hockey). As a result, the genes with the highest scientific background related to the sporting ability of athletes, namely angiotensin-converting enzyme (*ACE*), α-actinin-3 (*ACTN3*), nitric oxide synthase 3 (*NOS3*) and peroxisome proliferator-activator receptor alpha (*PPPARα*), are associated with performance in these modalities. However, further studies are needed to increase our knowledge (Zilberman-Schapira et al., 2012) because studies showing predisposition to injury are imperfect due the examination of one or a few candidate genes/polymorphisms on injury risk. Therefore, it would be interesting to extend this knowledge through the study of the combined influence of several genes/polymorphisms on injury risk in the same pool of athletes (Pitsiladis et al., 2016). In this sense, the knowledge of several DNA variants associated to injury may help to identify individuals with a greater predisposition to injury (Varillas-Delgado et al., 2021b).

In recent years, several genetic variants have been associated with an increased risk of sports-related injury or phenotypes that predispose athletes to injury. Among others, the adenosine monophosphate deaminase isoform 1 (*AMPD1*) (rs17602729), *ACE* (rs4646994), *ACTN3* (rs1815739), muscle-specific creatine kinase (*CKM*) (rs8111989) and creatine kinase light chain (*MLCK*; rs2849757 and rs2700352) are within the genes with the strongest scientific support (Lippi et al., 2010) to be linked to injury predisposition.

Previous research has shown that carriers of the T allele in the polymorphism of *AMPD1* c.34C>T (rs17602729) have reduced VO_2_max values and a lower response to endurance training (Lucia et al., 2009; Thomaes et al., 2011). The TT genotype is associated with metabolic myopathy, and exercise-induced muscle symptoms such as early fatigue, cramps and/or myalgia which are related to the risk of injury, especially in soccer (McCabe and Collins 2018). Also, some muscle-related metabolic diseases are related in individuals with the TT genotype (Gross 1997; Feng et al., 2017).

Thanks to recent advances in molecular biology and statistical genetics, it is possible to search for chromosomal regions containing genes predisposing to hypertension and to directly link specific mutations in candidate genes. Some of these markers are closely linked to genes of the renin-angiotensin system (Peters 1995). The most studied gene with the strongest association with hypertension is the *ACE* I/D polymorphism (rs4646994). Individuals with DD or ID genotypes are associated with the risk of hypertension in adulthood, compared with genotype II (Yan et al., 2018). This genetic variant in the *ACE* gene I/D has been recently associated with susceptibility to inflammation and muscle damage after exercise, showing that athletes with the II genotype are more susceptible to developing muscle injuries in professional soccer players (Massidda, Miyamoto-Mikami, Kumagai, Ikeda, Shimasaki, Yoshimura, Cugia, Piras, Scorcu, Kikuchi, Calò and Fuku 2020).

The *ACTN3* gene encodes the α-actinin-3, a highly conserved component of the contractile machinery of fast skeletal muscle fibers in mammals. The polymorphism c.1729C>T (rs1815739) affect the expression of α-actinin-3 in humans. Individuals with the CC genotype express functional α-actinin-3 and thig genotype is more frequent in athletes of power-oriented sports. On the contrary, the TT genotype (premature stop codon) is associated with complete α-actinin-3 deficiency, and athletes with this genotype are habitually more prone to muscle injury and exercise-induced muscle damage (Clarkson et al., 2005a; Clos et al., 2019; Massidda, Voisin, Culigioni, Piras, Cugia, Yan, Eynon and Calò 2019). The TT genotype reduces the diameter of fast fibers, increases the activity of multiple enzymes in the aerobic metabolic pathway and increases the activity of the aerobic metabolic pathway (Lippi et al., 2010). *ACTN3* has been related to susceptibility of injury in soccer, showing that the CC genotype reduces the incidence and severity of injuries with respect to the TT genotype in soccer players (Massidda, Voisin, Culigioni, Piras, Cugia, Yan, Eynon and Calò 2019).

A polymorphism in the 3′ non-coding region of *CKM* c.*800A>G (rs8111989) plays a vital role in the homeostatic energy of muscle cells and is responsible for the rapid regeneration of adenosine triphosphate (ATP) during intensive muscle contraction (Eider et al., 2015). Within this polymorphism, it has been found that the G allele reduces skeletal muscle activity during long-term efforts, with a lower concentration of creatine kinase (CK) (Zhou et al., 2006; Fedotovskaia et al., 2012).

*MLCK* gene polymorphisms c.37885C>A (rs28497577) and c.49C>T (rs2700352) alter the phosphorylation of regulatory light chains (RLC) and regulate the ability to produce force and resist tension during voluntary muscle contractions. Previous investigations have studied these polymorphisms under the hypothesis that they may predispose athletes to higher values of muscle damage during sports competitions (Del Coso et al., 2016; Mascarenhas et al., 2017) (Del Coso et al., 2016; Mascarenhas et al., 2017), showing that the C allele of c.37885C>A polymorphism (Del Coso, Valero, Lara, Salinero, Gallo-Salazar and Areces 2016) and T allele of c.49C>T (Clarkson et al., 2005) could predispose to higher values of muscle damage during exercise.

A recent study has shown that the sum of the influence of these genotypes has already been used to detect the likelihood of injury in other samples of athletes (Varillas-Delgado et al., 2022a) and we hypothesized that the characteristics of the injuries would show similarities with those found in elite endurance athletes with a high relevance of the *AMPD1* gene polymorphism in the risk of injuries etiology.

Therefore, this study aimed to examine, for the first time, the relationship between muscle injuries-related genes, risk and etiology of injuries in professional soccer players, and to demonstrate the feasibility of a total genotype score that correlates significantly with soccer injuries.

## Materials and methods

### Participants

This longitudinal cohort study analyzed 122 Spanish male professional soccer players in five clubs from LaLiga Santander and LaLiga Smartbank in Spain whose characteristics are presented in Table 1.

**TABLE 1:** Professional soccer players’ demographic characteristics.

		Total	Non-injured	Injured	p-value
Age, mean (SD)		23.43 (5.12)	22.96 (5.01)	23.76 (5.31)	0.405
Weight, mean (SD)		68.57 (7.26)	68.31 (6.25)	69.05 (7.96)	0.796
Height, mean (SD)		170.5 (5.68)	171.74 (5.28)	169.99 (6.08)	0.674
BMI, mean (SD)		23.42 (1.90)	22.95 (1.84)	24.32 (2.06)	0.156
Position	Goalkeepers, n (%)	14 (11.5)	10 (19.6)	4 (5.6)	0.074
	Defenders, n (%)	39 (32.0)	16 (31.4)	23 (32.4)	
	Midfielders, n (%)	33 (27.0)	14 (27.5)	19 (26.8)	
	Forwards, n (%)	36 (29.5)	11 (21.6)	25 (35.2)	

The following inclusion criteria were established for participants: a) professional soccer players with a contract with the first team of the football club; b) who participated in training and matches throughout the season at the same soccer club; c) performed regular exercise training of >1 h per day, > 5 days per week for the prior 6 months and; d) Spanish Caucasian descent for ≥3 generations (Varillas Delgado et al., 2019), and the exclusion criteria were: (a) contact injuries in the season; (b) incapacitating injuries for soccer training or matches in the previous 6 months to the onset of the study and; (c) professional female soccer players.

All players agreed to participate in the study by giving their written informed consent. The study protocol was approved by the research ethics committee of the Francisco de Vitoria University (UFV 32/2020) and the confidentiality of the participants was ensured, complying with the Declaration of Helsinki 1964 (latest update 2013).

### DNA sample collecting and genotype

The samples were collected during the 2021/2022 season in the months of March-May with SARSTED swabs by buccal smear and kept refrigerated until genotyping.

DNA extraction from the swabs was carried out in VIVOLabs laboratory (Madrid, Spain) by automatic extraction in QIACube equipment (QIAGEN, Venlo, Holland), yielding a DNA concentration of 25–40 ng/ml, which was kept in a solution in a volume of 100 μL at −20°C until genotyping.

*ACE* I/D (rs4646994), *ACTN3* c.1729C>T (rs1815739), *AMPD1* c.34C>T (rs17602729), *CKM* c.*800A>G (rs8111989) and *MLCK* c.37885C>A (rs28497577) and c.49C>T (rs2700352) polymorphisms were genotyped by using Single Nucleotide Primer Extension (SNPE) with the SNaPshot Multiplex Kit (Thermo Fisher Scientific, MA, United States), with analysis of the reaction result by capillary electrophoresis fragments, in an ABI3500 unit (Applied Biosystems, CA, United States) with bioinformatic analysis performed by GeneMapper 5.0 software (Applied Biosystems, CA, United States). Genomic location of each polymorphism is present in Table 2.

**TABLE 2:** Genomic location and minor allele frequency (MAF) for selected genes in muscle injuries.

Symbol	Gene	dbSNP	Genomic location	MAF soccer players	MAF (IBS)*	HWE	FIS
*ACE*	Angiotensin-converting enzyme	rs4646994	17q23.3	33.6% (I)	36.7% (I)**	*p* = 0.893	0.14
*ACTN3*	alpha-actinin-3	rs1815739	11q13.2	44.7% (T)	43.9% (T)	*p* = 0.842	−0.02
*AMPD1*	Adenosine monophosphate deaminase 1	rs17602729	1p13.2	20.8% (T)	14.0% (T)	*p* = 0.251	−0.48
*CKM*	Muscle-specific creatine kinase	rs8111989	19q13.32	33.2% (G)	26.6% (G)	*p* = 0.163	−0.25
*MLCK*	Myosin Light Chain Kinase	rs28497577	3q21.1	13.9% (A)	10.3% (A)	*p* = 0.621	−0.35
*MLCK*	Myosin Light Chain Kinase	rs2700352	3q21.1	22.1% (T)	20.1% (T)	*p* = 0.689	−0.10
*Overall SNPs*						*p* = 0.576	−0.18

IBS, Iberian population in Spain *(Yates et al., 2020) **(Fiuza-Luces et al., 2011); FIS, inbreeding coefficient; HWE, Hardy-Weinberg equilibrium; MAF, minor allele frequency; SNP, single nucleotide polymorphism.

### Injury collection

Baseline data were collected at the start of the season. Individual player exposure in training and matches was meticulously recorded by the clubs as part of their routines to estimate player load across the season. This research included the recording of all players with injuries and characteristics of all non-contact injuries reported by the medical services of the clubs during the 2021/2022 season. To be included in the analysis, the injury has to be the result of soccer exposure during either training or competition. Injuries caused due to a collision with another player or with an object were excluded from the investigation as they are potentially unaffected by the player’s genotype. The soccer injury doctor of each club recorded data on a paper player injury questionnaire. The questionnaire was completed when a soccer player required the attention of a doctor, and the results were sent to the head of medical services. The medical staff was informed that a recordable injury was defined as “any physical complaint suffered by a player as a result of a soccer match or training session, regardless of medical care or time lost from soccer activities”. Injuries collection followed the international consensus statement on procedures for epidemiological studies of soccer injuries recommended by the Fédération Internationale de Football Association (FIFA) and the Union of European Football Associations (UEFA) (Hägglund et al., 2005; Fuller et al., 2006; Bahr et al., 2020) including the etiological elements of the sports injury; 1) Rupture of muscle fibers 2) Onset of the injury (acute or gradual) and 3) Place of injury; if it occurred in a match or training session. Soccer injuries were classified using an adapted version of the *Orchard Sports Injury Classification* (Rae and Orchard 2007).

### Polygenic potential for muscle injuries

The combined influence of the six polymorphisms studied was calculated using a total genotype score (TGS) following the procedure presented by Williams and Folland (Williams and Folland 2008). A genotype score (GS) of 2 was assigned to the “protective” genotype for injuries, a GS of 1 was assigned to the heterozygous genotype while a GS of 0 was assigned to the “worst” genotype (Varillas-Delgado, et al., 2022a). The GS scores for the six polymorphisms in the professional soccer players cohort are shown in Table 3. The score of GS of all genotypes was transformed to a 0–100 arbitrary units (a.u) scale to facilitate interpretation, namely the total genotype score (TGS), as follows:

$$TGS=\left({GS}_{AMPD1}{+GS}_{ACE}{+GS}_{ACTN3}{+GS}_{CKM}{+GS}_{MLCK37885}{+GS}_{MLCK49}\right)\times 100/12$$

**TABLE 3:** Genotyping frequency in the professional soccer players.

Symbol	Gene	Polymorphism	dbSNP	Genotype score (Varillas Delgado et al., 2020b)	Professional football players
*ACE*	Angiotensin-converting enzyme	I/D	rs4646994	2 = DD	57 (46.8%)
				1 = ID	48 (39.3%)
				0 = II	17 (13.9%)
*ACTN3*	alpha-actinin-3	c.1729C>T	rs1815739	2 = CC	36 (29.5%)
				1 = CT	63 (51.6%)
				0 = TT	23 (18.9%)
*AMPD1*	Adenosine monophosphate deaminase 1	c.34C>T	rs17602729	2 = CC	86 (70.5%)
				1 = CT	24 (19.7%)
				0 = TT	12 (9.8%)
*CKM*	Muscle-specific creatine kinase	c.*800A>G	rs8111989	2 = GG	15 (12.3%)
				1 = GA	51 (41.8%)
				0 = AA	56 (45.9%)
*MLCK*	Myosin Light Chain Kinase	c.37885C>A	rs28497577	2 = AA	8 (6.6%)
				1 = CA	18 (14.8%)
				0 = CC	96 (78.6%)
	Myosin Light Chain Kinase	c.49T>C	rs2700352	2 = CC	74 (60.7%)
				1 = CT	42 (34.4%)
				0 = TT	6 (4.9%)

As previously indicated (Williams and Folland 2008), a TGS of 100 a.u. represents a “perfect protective” profile and a TGS of 0 a.u. would be the “worst protective” possible profile for muscle injuries when all GSs have a score of 0 a.u. Finally, the TGS distribution between non-injury and injury characteristics was assessed.

**FIGURE 1:**
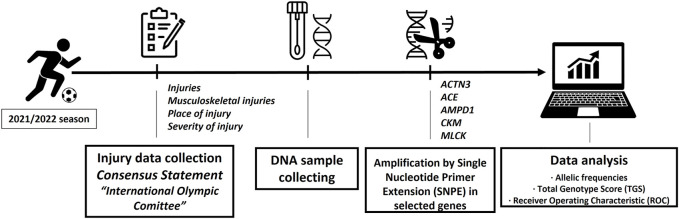
Experimental model.

The experimental model followed in this research is shown in Figure 1.

### Statistical analysis

Statistical analysis was performed using the Statistical Package for the Social Sciences (SPSS), v.21.0 for Windows (IBM Corp. Released 2012. IBM SPSS Statistics for Windows, Version 21.0. Armonk, NY: IBM Corp. United States).

Disequilibria of SNPs were estimated by using the Hardy-Weinberg Equilibrium (HWE) followed by the approach proposed by Weir and Cockerham (Weir and Hill 2002). The probability of having an optimal genotype for these polymorphisms between injured and non-injured was calculated using the χ2 test with a fixed α error of 0.05. The genotypic frequencies of the polymorphisms were compared between injury etiologies, using a χ2 test with fixed α 0.05. The ability of TGS to correctly distinguish injuries (0 = no, 1 = yes) and etiology; 1) musculoskeletal injuries (0 = no, 1 = yes), 2) place (0 = training, 1 = match) 3) and severity >28 days (0 = no, 1 = yes) was assessed using a receiver operating characteristic (ROC) curve (Zweig and Campbell 1993). Thus, the area under the ROC curve (AUC) was calculated with confidence intervals of 95% (95%CI). Finally, a binary logistic regression model was used to study the relationship between the TGS and etiology of soccer injuries.

## Results

The polymorphisms analyzed met the HWE (all *p* > 0.05; Table 2).

The characteristics of the injuries recorded and analyzed during the 2021/2022 season are presented in Table 4.

**TABLE 4:** Injury characteristics in professional soccer players during the 2021/2022 season.

		Frequency
Onset	Acute	107 (88.4%)
	Repetitive/overuse	14 (11.6%)
Place	Training	60 (49.6%)
	Match	61 (50.4%)
Tissue affected	Bone	2 (1.7%)
	Joints and ligaments	51 (42.1%)
	Muscles and tendons	66 (54.5%)
	Skin	0 (0.0%)
	Nervous system	1 (0.8%)
	Other	1 (0.8%)
Severity	Slight	26 (21.5%)
	Mild	13 (10.7%)
	Moderate	56 (46.3%)
	Severe	26 (21.5%)
Type	Fracture	0 (0.0%)
	Other bone injuries	2 (1.7%)
	Luxation/subluxation	4 (3.3%)
	Ligamentous distension	29 (24.0%)
	Ligamentous tear	4 (3.3%)
	Meniscus injury	4 (3.3%)
	Chondral injury	2 (1.7%)
	Synovitis	1 (0.8%)
	Fasciitis	1 (0.8%)
	Muscle tear	54 (44.6%)
	Muscle contracture	8 (6.6%)
	Tendon strain	6 (5.0%)
	Tendon rupture	2 (1.7%)
	Bursitis	1 (0.8%)
	Tendinitis	1 (0.8%)
	Hematoma-contusion	0 (0.0%)
	Abrasion	0 (0.0%)
	Incised wound	0 (0.0%)
	Concussion	0 (0.0%)
	Dental injuries	0 (0.0%)
	Nerve injury	1 (0.8%)
	Other injuries	1 (0.8%)
Recurrency	No	105 (86.8%)
	Yes	16 (113.2%)

### Injuries

Of the 122 professional soccer players, 71 players had a non-contact injury during the season (58.2%) for a total of 121 injuries. The remaining 51 players (41.8%) did not present any injury throughout the 2021–2022 season.

When adding the genotype scores of all polymorphisms, the mean value of the TGS in the non-injured players was 57.18 a.u. (±14.43 a.u.), statistical kurtosis: 0.32 (±0.24). The TGS value for the group of injured players was 51.71 a.u. (±12.82 a.u.), statistical kurtosis: 0.38 (±0.32). The TGS values between the non-injured and injured professional soccer players were statistically significant (*p* = 0.034).

**FIGURE 2:**
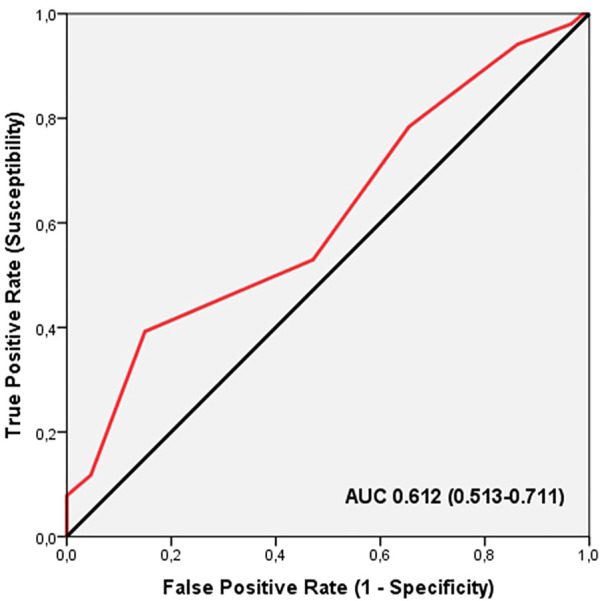
Receiver operating characteristic curve (ROC) summarizing the ability of the total genotype score (TGS) to distinguish potential non-injured players from injured players in muscle performance profile, AUC, area under the curve.

ROC analysis showed significant discriminatory accuracy of TGS in the identification of injuries in professional soccer players (AUC = 0.612; 95%CI: 0.513–0.711; *p* = 0.028) (sensitivity = 0.640, specificity = 0.475) (Figure 2). The corresponding TGS value at this point was 45.83 a.u. Binary logistic regression analysis showed that subjects with a TGS lower than 45.83 a.u. had an odds ratio (OR) of 1.91 (95%CI: 1.14–2.91; *p* = 0.022) of injury during the season, compared to those with a TGS above this value.

Genotype distribution of muscle performance genes in the non-injured professional soccer players group, when compared with the injured players, was statistically significant for *AMPD1* polymorphism (*p* < 0.001), showing a higher frequency in the “optimal” genotype in non-injured players (GG 88.2%) than the injured players (GG 57.7%), and the injured players, showing a higher frequency of the “worse” genotype (TT 15.5%) than the non-injured players (TT 2.0%) (Table 5). Also, statistical differences were found for *MLCK* c.37885C>A polymorphism (*p* = 0.003), showing a higher frequency of the “optimal” genotype in non-injured players (AA 15.7%) than in injured players (AA 0.0%), as well as injured players showing a higher frequency of the “worse” genotype (CC 84.5%) than non-injured players (CC 70.6%) (Table 5). No differences were shown in the other muscle performance polymorphisms between non-injured and injured players (Table 5).

**TABLE 5:** Genotype distribution in non-injured and injured professional soccer players for muscle injuries polymorphisms.

Symbol	Gene	Polymorphism	dbSNP	Genotype score (Varillas Delgado et al., 2020a)	Non-injured professional soccer players	Injured professional soccer players	*p*-value
*ACE*	Angiotensin-converting enzyme	I/D	rs4646994	2 = DD	27 (52.9%)	30 (42.3%)	0.311
				1 = ID	16 (31.4%)	32 (45.0%)	
				0 = II	8 (15.7%)	9 (12.7%)	
*ACTN3*	alpha-actinin-3	c.1747C>T	rs1815739	2 = CC	15 (29.4%)	21 (29.6%)	0.250
				1 = CT	23 (45.1%)	40 (56.3%)	
				0 = TT	13 (25.5%)	10 (14.1%)	
*AMPD1*	Adenosine monophosphate deaminase 1	c.34C>T	rs17602729	2 = CC	45 (88.2%) ↑	41 (57.7%) ↓	<0.001
				1 = CT	5 (9.8%) ↓	19 (26.8%) ↑	
				0 = TT	1 (2.0%) ↓	11 (15.5%) ↑	
*CKM*	Muscle-specific creatine kinase	c.*800A>G	rs8111989	2 = GG	8 (15.7%)	7 (9.9%)	0.387
				1 = GA	23 (45.1%)	28 (39.4%)	
				0 = AA	20 (39.2%)	36 (50.7%)	
*MLCK*	Myosin Light Chain Kinase	c.37885C>A	rs28497577	2 = AA	8 (15.7%) ↑	0 (0.0%) ↓	0.003
				1 = CA	7 (13.7%)	11 (15.5%)	
				0 = CC	36 (70.6%) ↓	60 (84.5%) ↑	
	Myosin Light Chain Kinase	c.49T>C	rs2700352	2 = CC	31 (60.8%)	43 (60.6%)	0.906
				1 = CT	18 (35.3%)	24 (33.8%)	
				0 = TT	2 (3.9%)	4 (5.6%)	

### Musculoskeletal injuries

Seventy-one players had 121 non-contact injuries during the season of which 54 (44.6%) were musculoskeletal injuries.

In the genotype scores of all polymorphisms, the mean value of players with non-musculoskeletal injured has a TGS of 51.11 a.u. (±11.32 a.u.), statistical kurtosis: 0.41 (±0.31). The value for the group of musculoskeletal injured players was 46.75 a.u. (±12.14 a.u.), statistical kurtosis: 0.28 (±0.29). The TGS values between non-musculoskeletal injured and injured professional soccer players were statistically significant (*p* = 0.047).

**FIGURE 3:**
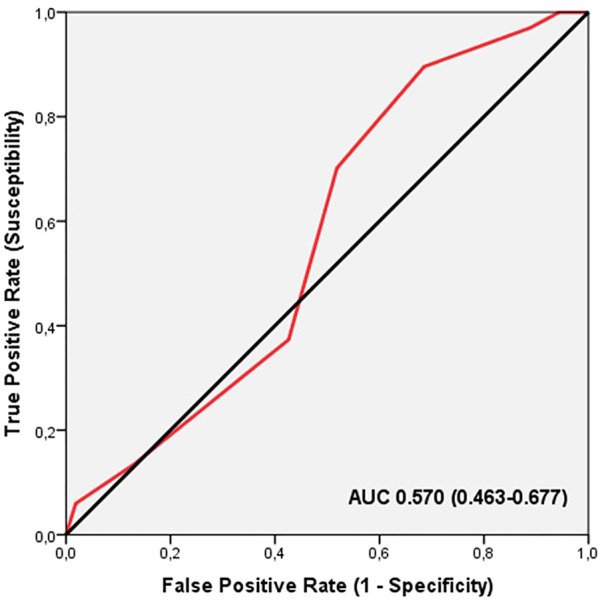
Receiver operating characteristic curve (ROC) summarizing the ability of the total genotype score (TGS) to distinguish potential musculoskeletal non-injured players from musculoskeletal injured players in muscle performance profile, AUC, area under the curve.

ROC analysis did not show significant discriminatory accuracy of TGS in the identification of musculoskeletal injuries in professional soccer players (AUC = 0.570; 95%CI: 0.463–0.677; *p* = 0.189) (sensitivity = 0.373, specificity = 0.426) (Figure 3). The corresponding TGS value at this point was 54.16 a.u. Binary logistic regression analysis showed that subjects with a TGS lower than 54.16 a.u. had an odds ratio (OR) of 1.25 (95%CI: 0.60–2.59; *p* = 0.555) of musculoskeletal injury during the season, compared to those with a TGS above this value.

However, the genotype distribution of muscle performance genes in players in the non-musculoskeletal injury group, when compared to musculoskeletal injured players, was statistically significant for the *AMPD1* polymorphism (*p* = 0.004), showing a higher frequency in the “optimal” genotype in non-musculoskeletal injured players (GG 53.7%) than the musculoskeletal injured players (GG 25.9%), with musculoskeletal injured players having a higher frequency of the “worse” genotype (TT 31.5%) than non-musculoskeletal injured players (TT 13.4%) (Table 6). Regarding the *CKM* polymorphism, differences were observed in the GA genotype, with a higher frequency in players with a musculoskeletal injury (GA 42.3%) than in players without a musculoskeletal injury (GA 25.4%) (Table 6). No differences were shown in the other muscle performance polymorphisms between non-injured and injured players (Table 6).

**TABLE 6:** Genotype distribution in professional soccer players with non-musculoskeletal and musculoskeletal injuries for muscle injuries polygenic profile.

Symbol	Gene	Polymorphism	dbSNP	Genotype score (Varillas Delgado et al., 2020a)	Non-musculoskeletal injured professional soccer players	Musculoskeletal injured professional soccer players	*p*-value
*ACE*	Angiotensin-converting enzyme	I/D	rs4646994	2 = DD	24 (35.8%)	17 (31.5%)	0.583
				1 = ID	34 (50.7%)	26 (48.1%)	
				0 = II	9 (13.5%)	11 (20.4%)	
*ACTN3*	alpha-actinin-3	c.1747C>T	rs1815739	2 = CC	21 (31.3%)	18 (33.3%)	0.972
				1 = CT	36 (53.7%)	28 (51.9%)	
				0 = TT	10 (14.95)	8 (14.8%)	
*AMPD1*	Adenosine monophosphate deaminase 1	c.34C>T	rs17602729	2 = CC	36 (53.7%) **↑**	14 (25.9%) ↓	0.004
				1 = CT	22 (32.8%)	23 (42.6%)	
				0 = TT	9 (13.4%) ↓	17 (31.5%) ↑	
*CKM*	Muscle-specific creatine kinase	c.*800A>G	rs8111989	2 = GG	7 (10.4%)	5 (9.3%)	0.131
				1 = GA	17 (25.4%) ↓	23 (42.3%) ↑	
				0 = AA	43 (64.2%)	26 (48.1%)	
*MLCK*	Myosin Light Chain Kinase	c.37885C>A	rs28497577	2 = AA	0 (0.0%)	0 (0.0%)	0.475
				1 = CA	16 (23.9%)	10 (18.5%)	
				0 = CC	51 (76.1%)	44 (81.5%)	
	Myosin Light Chain Kinase	c.49T>C	rs2700352	2 = CC	44 (65.6%)	33 (61.1%)	0.471
				1 = CT	18 (26.9%)	19 (35.2%)	
				0 = TT	5 (7.5%)	2 (3.7%)	

### Place of injury

Of the 121 non-contact injuries analyzed, 61 (50.4%) occurred in matches, compared to 60 (49.6%) in training.

In the analysis of the genotype scores of all polymorphisms, the mean value of players injured during a match had a TGS of 50.27 a.u. (±11.48 a.u.), statistical kurtosis: 0.28 (±0.31). The value for injuries occurring in training was 48.05 a.u. (±14.66 a.u.), statistical kurtosis: 0.43 (±0.37). The TGS values between match and training injuries were not statistically significant (*p* = 0.356).

**FIGURE 4:**
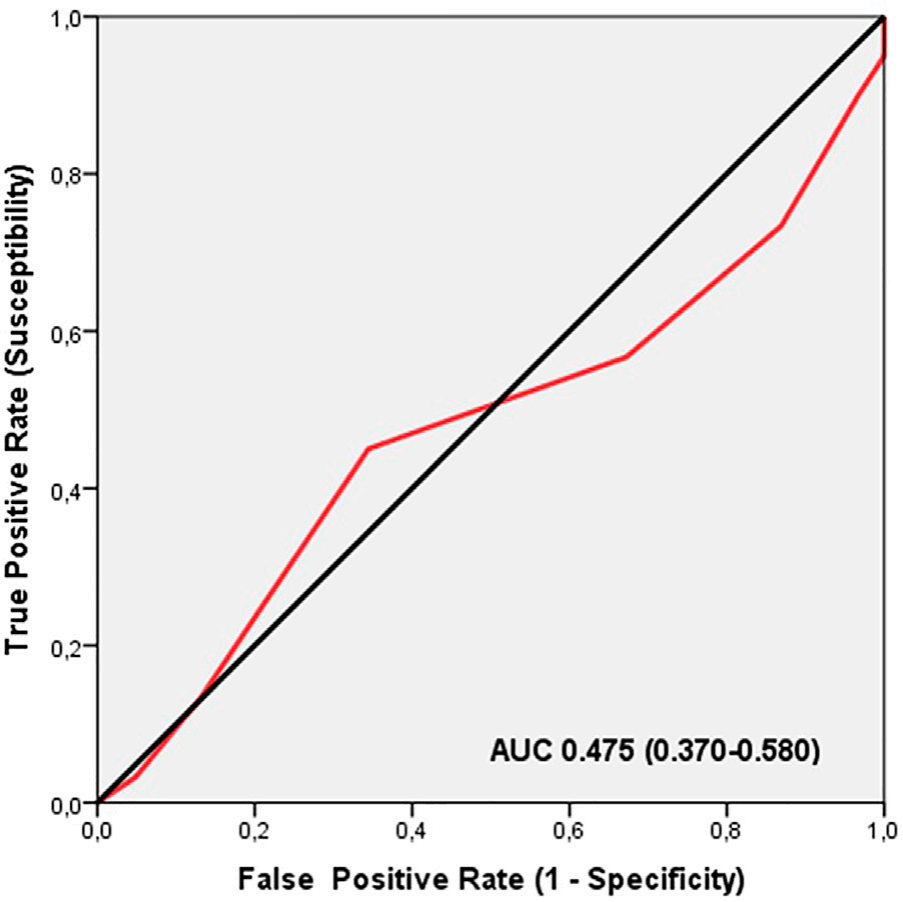
Receiver operating characteristic curve (ROC) summarizing the ability of the total genotype score (TGS) to distinguish potential match injured players from training injured players in muscle performance profile, AUC, area under the curve.

ROC analysis did not show significant discriminatory accuracy of TGS in the identification of place of injuries in professional soccer players (AUC = 0.475; 95%CI: 0.370–0.580; *p* = 0.635) (sensitivity = 0.450, specificity = 0.344) (Figure 4). The corresponding TGS value at this point was 54.16 a.u. Binary logistic regression analysis showed that subjects with a TGS lower than 54.16 a.u. had an odds ratio (OR) of 1.58 (95%CI: 0.89–2.36; *p* = 0.236) of match injuries, compared to those with a TGS above this value.

Genotype distribution of muscle performance genes in players with match injuries, when compared to players with training injuries was statistically significant for the *MLCK* c.37885C>A polymorphism (*p* = 0.009), showing a higher frequency of “worse” genotype (CC 88.3%) in training injured players than in match injured players (CC 68.9%), while in the CA genotype, differences between frequencies were found, with a higher frequency in matches injured players (CA 31.1%) than in training injured players (CA 11.7%) (Table 7). Regarding the *CKM* polymorphism, differences were observed in the GG genotype, with a higher frequency in players with training injuries (GG 15.0%) than in players with match injuries (GG 4.9%) (Table 7). No differences were shown in the other muscle performance polymorphisms between non-injured and injured players (Table 7).

**TABLE 7:** Genotype distribution in professional soccer players with matches and training injuries for muscle injuries polygenic profile.

Symbol	Gene	Polymorphism	dbSNP	Genotype score (Varillas Delgado et al., 2020b)	Training injured professional soccer players	Match injured professional soccer players	*p*-value
*ACE*	Angiotensin-converting enzyme	I/D	rs4646994	2 = DD	21 (35.0%)	20 (32.8%)	0.959
				1 = ID	29 (48.3%)	31 (50.8%)	
				0 = II	10 (16.7%)	10 (16.4%)	
*ACTN3*	alpha-actinin-3	c.1747C>T	rs1815739	2 = CC	16 (26.7%)	23 (37.7%)	0.291
				1 = CT	36 (60.0%)	28 (45.9%)	
				0 = TT	8 (13.3%)	10 (16.4%)	
*AMPD1*	Adenosine monophosphate deaminase 1	c.34C>T	rs17602729	2 = CC	25 (41.7%)	25 (41.0%)	0.119
				1 = CT	18 (30.0%)	27 (44.3%)	
				0 = TT	17 (28.3%)	9 (14.8%)	
*CKM*	Muscle-specific creatine kinase	c.*800A>G	rs8111989	2 = GG	9 (15.0%) ↑	3 (4.9%) ↓	0.178
				1 = GA	19 (31.7%)	21 (34.4%)	
				0 = AA	32 (53.3%)	37 (60.7%)	
*MLCK*	Myosin Light Chain Kinase	c.37885C>A	rs28497577	2 = AA	0 (0.0%)	0 (0.0%)	0.009
				1 = CA	7 (11.7%) ↓	19 (31.1%) ↑	
				0 = CC	53 (88.3%) ↑	42 (68.9%) ↓	
	Myosin Light Chain Kinase	c.49T>C	rs2700352	2 = CC	38 (63.3%)	39 (63.9%)	0.916
				1 = CT	19 (31.7%)	18 (29.5%)	
				0 = TT	3 (5.0%)	4 (6.6%)	

### Severity of injury

Of the 121 non-contact injuries analyzed, 26 (21.5%) were severe (>28 days), while 95 injuries (78.5%) were non-severe (<28 days).

In the analysis of the genotype scores of all polymorphisms, the mean value of players with severe injuries had a TGS of 49.67 a.u. (±12.35 a.u.), statistical kurtosis: 0.33 (±0.26). The value for non-severe injuries was 49.03 a.u. (±13.41 a.u.), statistical kurtosis: 0.30 (±0.29). The TGS values between non-severe and severe injuries were not statistically significant (*p* = 0.826).

**FIGURE 5:**
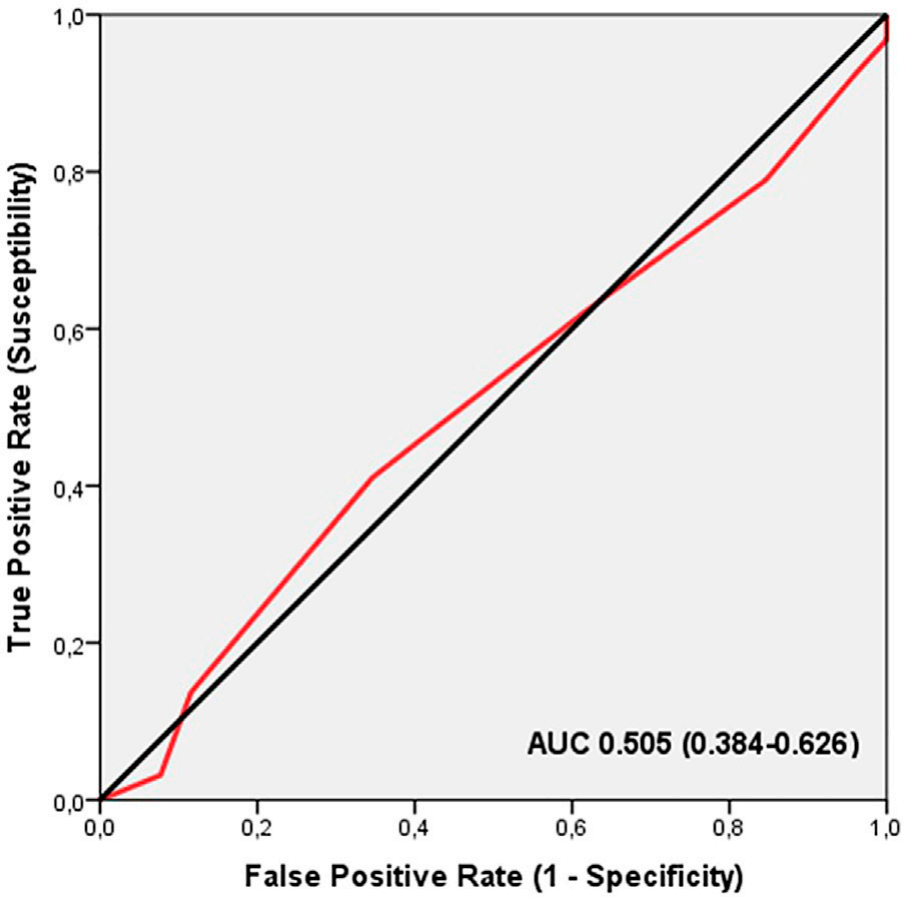
Receiver operating characteristic curve (ROC) summarizing the ability of the total genotype score (TGS) to distinguish potential non-severely injured players from severely injured players in muscle performance profile, AUC, area under the curve.

ROC analysis did not show significant discriminatory accuracy of TGS in the identification of severity of injuries in professional soccer players (AUC = 0.505; 95%CI: 0.384–0.626; *p* = 0.942) (sensitivity = 0.411, specificity = 0.346) (Figure 5). The corresponding TGS value at this point was 54.16 a.u. Binary logistic regression analysis showed that subjects with a TGS lower than 54.16 a.u. had an odds ratio (OR) of 1.31 (95%CI: 0.45–3.63; *p* = 0.553) of severe injuries, compared to those with a TGS above this value.

The genetic distribution in the muscle performance genes when compared between players who had a severe injury and those who had a non-severe injury, showed no differences in the polymorphisms studied. However, in the *ACTN3* gene, it was observed that the TT genotype showed a higher frequency in players with severe injuries (TT 26.9%) than in non-severe injuries (TT 11.6%) (Table 8). Regarding the *CKM* polymorphism, differences were observed in the GA genotype, with a higher frequency in players with a severe injury (GA 50.0%) than in players with a non-severe injury (GA 28.4%) (Table 8).

**TABLE 8:** Genotype distribution in professional soccer players with non-severe and severe injuries for muscle injuries polygenic profile.

Symbol	Gene	Polymorphism	dbSNP	Genotype score (Varillas Delgado et al., 2020a)	Non-severely injured professional soccer players	Severely injured professional soccer players	*p*-value
*ACE*	Angiotensin-converting enzyme	I/D	rs4646994	2 = DD	32 (33.7%)	9 (34.6%)	0.366
				1 = ID	45 (47.4%)	15 (57.7%)	
				0 = II	18 (18.9%)	2 (7.7%)	
*ACTN3*	alpha-actinin-3	c.1747C>T	rs1815739	2 = CC	32 (33.7%)	7 (26.9%)	0.149
				1 = CT	52 (54.7%)	12 (46.2%)	
				0 = TT	11 (11.6%) ↓	7 (26.9%) ↑	
*AMPD1*	Adenosine monophosphate deaminase 1	c.34C>T	rs17602729	2 = CC	36 (37.9%)	14 (53.8%)	0.333
				1 = CT	37 (38.9%)	8 (30.8%)	
				0 = TT	22 (23.2%)	4 (15.4%)	
*CKM*	Muscle-specific creatine kinase	c.*800A>G	rs8111989	2 = GG	11 (11.6%)	12 (46.2%)	0.091
				1 = GA	27 (28.4%) ↓	13 (50.0%) ↑	
				0 = AA	57 (60.0%)	1 (3.8%)	
*MLCK*	Myosin Light Chain Kinase	c.37885C>A	rs28497577	2 = AA	0 (0.0%)	0 (0.0%)	0.752
				1 = CA	21 (22.1%)	5 (19.2%)	
				0 = CC	74 (77.9%)	21 (80.8%)	
	Myosin Light Chain Kinase	c.49T>C	rs2700352	2 = CC	62 (65.3%)	15 (57.7%)	0.585
				1 = CT	27 (28.4%)	10 (38.5%)	
				0 = TT	6 (6.3%)	1 (3.8%)	

## Discussion

Although the influence of several polymorphisms on exercise performance and the likelihood of injury have been analyzed in several previous investigations, to the authors’ knowledge, this is the first to investigate the association between muscle injury-related genes and the likelihood of injury in professional soccer players. The purpose of this study was to examine the relationship between muscle injury-related genes, injury risk and injury etiology in professional soccer players. The main finding in this study is the correlation between TGS scores and different injury characteristics, with the C allele of the *AMPD1* polymorphism suggesting some type of injury prevention-related benefit, and especially to protect against musculoskeletal injury.

The main outcome of this research indicates that injury incidence was similar in professional soccer players with the *ACE*, *ACTN3*, *CKM* and *MLCK* c.49T>C polymorphisms (Table 5). However, players with different *AMPD1* and *MLCK* c.37885C>A genotypes showed injury incidence with different characteristics: (a) players with the TT genotype of the *AMPD1* gene had a higher frequency of injuries, with a higher frequency of musculoskeletal injuries compared to the CC genotype; (b) players with the CC genotype of the *MLCK* c.37885C>A polymorphism had a higher frequency of injuries, especially during training. The CA genotype showed a higher frequency of injuries in matches, while the AA genotype suggests some injury-related benefit throughout the season; (c) the GA genotype of the *CKM* gene showed a higher frequency of musculoskeletal injuries in professional soccer players, as well as in the severity of injuries occurring in the season. Players with the GG genotype had a higher frequency of injuries in training than in matches and; (d) players with the TT genotype of the *ACTN3* gene had a higher frequency of severe injuries. Collectively, this information suggests that *AMPD1*, *CKM*, *MLCK* c.37885C>A and *ACTN3* genotypes affect the likelihood of non-contact injuries during the soccer season. Furthermore, having the CC genotype at the *AMPD1* polymorphism is somewhat protective against injuries, and especially musculoskeletal injuries, as occurs with the AA genotype at the *MLCK* c.37885C>A polymorphism, with the CC genotype recording more frequent injuries in training than the CA genotype, while higher frequencies of severe injuries occurred in TT players at the *ACTN3* gene, while the GA genotype of the *CKM* gene had a higher frequency of musculoskeletal injuries.

Injuries are a common problem in soccer and are influenced by both player’s intrinsic factors and environmental variables. Among others, training methods and injury prevention programs may condition injury risk and they may be a confounding factor for this study. However, we selected a homogeneous sample of professional soccer players while the clubs involved in this study had similar training methodologies and comparable competitive load throughout the season. For example, all clubs included strength training with external loads >1 time per week, proprioceptive training, and multicomponent programs (balance, core stability, and functional strength and mobility) to prevent injury. Despite the similar characteristics of training and competition load for all the players involved in this study, only 16.6% suffered a non-contact injury. The current investigation reflects that genetics may constitute an important contributing factor for a player’s predisposition to injury. Additionally, through the assessment of a TGS that includes only 6 SNPs, players with a higher predisposition to injury can be identified and placed into a special program to reduce their nature-associated susceptibility to injury. The knowledge provided by this research with genetic data could constitute a new tool to predict a higher risk of injury in professional soccer players alongside established training methodologies.

Although excellent muscle performance in sports is facilitated by an optimal polygenic profile (Egorova et al., 2014; Mohr et al., 2016; Jacob et al., 2019; Pickering et al., 2019; McAuley et al., 2021a), the methodological rigor and evidence in genetic association research in soccer still has room for improvement (McAuley, Hughes, Tsaprouni, Varley, Suraci, Roos, Herbert and Kelly 2021a). This analysis indicates that the combined influence of these five key genes is strong enough to discriminate the risk of soccer injuries. Previous research has focused on several genes and their influence in the response to physical training and predisposition to soccer-related injuries, especially in *ACTN3* (Coelho et al., 2018; Clos, Pruna, Lundblad, Artells and Esquirol Caussa 2019; Massidda, Voisin, Culigioni, Piras, Cugia, Yan, Eynon and Calò 2019; Clos et al., 2021) and *ACE* polymorphisms (Massidda, Miyamoto-Mikami, Kumagai, Ikeda, Shimasaki, Yoshimura, Cugia, Piras, Scorcu, Kikuchi, Calò and Fuku 2020; McAuley et al., 2021b; Wei 2021). Identification of genetic markers associated with the regulation of energy metabolism in skeletal muscles can help sports physicians and coaches develop personalized strategies to adapt the training protocols according to this professional soccer player’s genetic profile presented (Varillas-Delgado et al., 2022b).

*AMPD1* is an important regulator of energy metabolism in the muscle fiber that shifts the equilibrium of the reaction of the purine nucleotide cycle towards adenosine triphosphate production by converting adenosine monophosphate into inosine monophosphate (Maciejewska-Skrendo et al., 2019). Individuals with the TT genotype have diminished exercise capacity and cardiorespiratory responses to exercise (Rico-Sanz et al., 2003; Varillas Delgado et al., 2020). Moreover, carriers of the T allele have a limited training response of ventilatory phenotypes during maximal exercise (Rico-Sanz, Rankinen, Joanisse, Leon, Skinner, Wilmore, Rao and Bouchard 2003; Rubio et al., 2005; Gineviciene et al., 2014) and a reduced submaximal aerobic capacity (Lucia et al., 2009; Gineviciene, Jakaitiene, Pranculis, Milasius, Tubelis and Utkus 2014), showing an inability to catalyze the purine nucleotide cycle reaction, preventing the muscle cells from having energy for their function, leading to their death, which is the cause of the injury, especially musculoskeletal injury, discovered in this research. This study shows that the *AMPD1* gene is a relevant factor for soccer injuries, especially the ones with a musculoskeletal nature. These data are similar to those recently published on injuries in elite endurance athletes (Varillas-Delgado et al., 2022b). Collectively, this information suggests that optimal muscle function -through optimal genotype in key genes associated to muscle function-influences injury in professional soccer players. These results are valuable for future studies to demonstrate a cause of muscle performance and injuries that could lead to knowledge in this field of sports injuries.

*CKM* plays an important role in energy provision during high-intensity muscle contraction. The main outcomes of this research indicate that injury incidence was similar in soccer players, with data like those presented in our study (Table 5). The GA genotype is associated with a greater propensity for fiber rupture and injury severity than GG or AA players, which could lead to the conclusion that the GG and AA genotypes may offer a protective role. This may be of particular interest for soccer practitioners to prevent muscle tears and their severity during professional soccer practice as this type of injury is the most common at the elite level (López-Valenciano et al., 2020).

*MLCK* plays a critical role in the regulation of smooth muscle contraction (Stull et al., 2011; Sun et al., 2020). In this gene, two polymorphisms have been associated with post-exercise strength loss (Shen et al., 2015; Del Coso, Valero, Lara, Salinero, Gallo-Salazar and Areces 2016), showing that heterozygotes CA for c.37885C>A might present higher exercise-induced muscle damage after an endurance competition than CC counterparts (Clarkson et al., 2005a). These data are in agreement with those presented in this research in which the CA genotype is associated with a higher incidence of injuries in matches, where the muscle works at a maximum level, in continuous endurance efforts, while the CC genotype is shown to have a higher incidence of injuries from repetitive endurance efforts that occur during the course of a professional soccer match (Table 7). In turn, it was observed that the AA genotype has a protective benefit against injuries in professional soccer players, with the CC genotype prevalent in 84.5% of injuries compared to 70.6% of players with the AA genotype (Table 5). These results show that optimal muscle contraction and reduced strength loss during exercise are associated with the AA and CA genotypes, with the A allele showing an association with strength preservation during sporting efforts; data that will be confirmed in further studies in this same cohort of athletes.

There is some evidence that genetic variation in the *ACE* gene might be associated with many heritable traits, including physical performance (Moran et al., 2006) and injuries in professional soccer players (Massidda, Miyamoto-Mikami, Kumagai, Ikeda, Shimasaki, Yoshimura, Cugia, Piras, Scorcu, Kikuchi, Calò and Fuku 2020). Specifically, there is an increased frequency of injuries in the I allele in Italian professional soccer players, which could influence the susceptibility to develope muscle injuries among soccer players. The D allele has been associated with elite sprint performance (Myerson et al., 1999), and with an improvement of explosive strength, especially in countermovement jump (CMJ) and sprint tests (Melián Ortiz et al., 2021). However, in this study, *ACE* genotypes have not been related to the risk of injury or to the aspects of etiology analyzed. The *ACE* gene has been studied as a marker associated with soccer player status by genetic scoring using the TGS, along with other genes, such as *ACTN3* (Egorova, Borisova, Mustafina, Arkhipova, Gabbasov, Druzhevskaya, Astratenkova and Ahmetov 2014). In this case, the D allele has been shown to be a predictor of elite soccer player status (McAuley, Hughes, Tsaprouni, Varley, Suraci, Roos, Herbert and Kelly 2021b); data that should be corroborated by future studies to confirm this gene as a predictor of elite athlete status.

The c.1729C>T polymorphism of the *ACTN3* gene is a highly conserved component of the contractile machinery in fast skeletal muscle fibers in mammals (Lippi et al., 2010; Eynon, Hanson, Lucia, Houweling, Garton, North and Bishop 2013), with the CC genotype present among elite power athletes, whereas the TT genotype, associated with complete *ACTN3* deficiency, is more prevalent among elite endurance athletes, such as marathon runners and cyclists (Yang et al., 2003). The *ACTN3* gene has been extensively studied and associated with injuries in elite athletes (Del Coso et al., 2019; Zouhal et al., 2021) in athletics (Moreno et al., 2020; Gutiérrez-Hellín et al., 2021), ballerinas (Kim et al., 2014) and especially in soccer (Coelho, Pimenta, Rosse, de Castro, Becker, de Oliveira, Carvalho and Garcia 2018; Clos, Pruna, Lundblad, Artells and Esquirol Caussa 2019; Massidda, Voisin, Culigioni, Piras, Cugia, Yan, Eynon and Calò 2019; Rodas et al., 2021). Although these studies suggest that the TT genotype (erroneously referred to as the XX genotype (Dunnen et al., 2016)) is associated with an increased risk of injury, there is still conflicting data, and this gene has not been shown to have a direct link to sports injuries. With the data presented in this study, the authors conclude that the cause of injuries in soccer players is due more to the inability of muscle cells to produce energy because of the c.34C>T polymorphism of the *AMPD1* gene that causes cell death than to the composition of muscle fibers has recently been demonstrated (Varillas-Delgadoet al 2022a). However, it has been shown that the TT genotype is associated in professional soccer players with greater injury severity as previously presented in the study by Massidda et al. (Massidda, Voisin, Culigioni, Piras, Cugia, Yan, Eynon and Calò 2019). Although other studies do not show the association of the TT genotype with the severity of these injuries in professional soccer players (Clos, Pruna, Lundblad, Artells and Esquirol Caussa 2019), further studies of injuries in this cohort of athletes are needed to confirm the results found. Finally, this study does not demonstrate the influence of the *ACTN3* gene on the risk of injury in professional soccer players.

This information on the six polymorphisms implicated in muscular performance shows that the likelihood of sustaining an injury in professional soccer players was higher in the TGS <45.83 a.u. group than in their >45.83 a.u. counterparts. However, in the probability of suffering musculoskeletal injuries, site of injury and severity, no discriminatory point was found in the TGS at which a cut-off point could be defined in these etiological characteristics of injuries in soccer players. The study of a single polymorphism with an injury phenotype should be avoided in the field of genetics in the future (Yang, MacArthur, Gulbin, Hahn, Beggs, Easteal and North 2003; Pickering and Kiely 2017; McAuley, Hughes, Tsaprouni, Varley, Suraci, Roos, Herbert and Kelly 2021b), and studies such as the one presented in this investigation using TGS should be implemented. The design of specific programs to prevent muscle damage and injury in soccer players using this genetic profile merits further investigation.

This model presents some limitations: a) although we have already manifested that the training routines and competition load of the clubs recruited for this investigation was comparable, it is still possible that the intrinsic characteristics of other training aspects as tactical exercises or the game style used by the teams had influenced the results of this study; b) the sample size of the group of professional soccer players was limited, but the cohort presented in this study is a representative sample; c) numerous genetic variants that have not been included in the model are likely to appear in the foreseeable future, that can also explain individual variations in the potential for attaining professional soccer performance and injuries, and; d) more longitudinal studies of large cohorts of professional soccer players including women players are necessary before the evidence linking the genetics and epidemiology of injuries can be established, and it is still premature to use genetic testing among professional soccer players to effectively predict the risk of injury.

Further study may help to develop this as a potential tool to help predict injury risk in these soccer players. In fact, the genes with a positive association with muscle performance and protection against injuries were the *AMPD1* and *MLCK* c.37885C>A polymorphisms. *AMPD1* also showed protection against musculoskeletal injuries, presenting itself as a key gene in the new knowledge of injuries in soccer due to its muscle energy optimizing functions (Varillas Delgado et al., 2020a; Varillas Delgado et al., 2020b). Lastly, the current investigation raises the need to include epigenetics and environmental aspects in the analysis of the factors associated with soccer performance according to muscle metabolism, improving the understanding of the links between genetics and injuries (Varillas-Delgado et al., 2022b).

## Conclusion

This study is the first to demonstrate that injury incidence in professional soccer players was affected by a TGS obtained the combination of favorable/unfavorable genotypes in genes involved in muscle performance. The TT genotype and T allele of *AMPD1* appears to be the polymorphism that best correlates with a higher risk of injury in this cohort of professional soccer players.

## Data Availability

All relevant data are within the paper. All manuscript files are available from the Research Square database (access number 10.21203/rs.3.rs-1296652/v1).
